# A novel approach to managing gingival recession in protruded roots: a case report

**DOI:** 10.1093/jscr/rjaf044

**Published:** 2025-02-12

**Authors:** Cezar Lahham, Rand Kharroubi, Basem AbuQubi, Aram Shatara, Elias Edward Lahham

**Affiliations:** Department of Periodontology, Arab American University, PO Box 240, 13 Zababdeh, Jenin, Palestine; Department of Periodontology, Al-Quds University, Abu-Dis, Palestinian Authority, East Jerusalem, Palestine; Department of Periodontology, Al-Quds University, Abu-Dis, Palestinian Authority, East Jerusalem, Palestine; Department of Periodontology, Al-Quds University, Abu-Dis, Palestinian Authority, East Jerusalem, Palestine; Department of Radiation Oncology, Augusta Victoria Hospital, Martin Buber Jerusalem 9119101, Palestinian Authority, East Jerusalem, Palestine

**Keywords:** odontoplasty, gingival recession, connective tissue graft, root surface biomodification

## Abstract

This case report presents an innovative approach to managing severe isolated gingival recession associated with root protrusion beyond bone boundaries in a 28-year-old female patient. The patient, who had a history of orthodontic treatment for a 5-year-period, displayed significant gingival recession at the lower right central incisor. Conventional orthodontic repositioning was declined by the patient, leading to the adoption of an alternative treatment strategy. A combination of odontoplasty to trim the protruding root (~1 mm) and a connective tissue graft was employed. The procedure involved meticulous flap creation, root surface biomodification, and precise graft placement to achieve optimal functional and esthetic outcomes. Follow-up over 3 months revealed increased keratinized tissue thickness and complete root coverage, with high patient satisfaction. This novel technique demonstrates the potential to address complex recession cases effectively, preserving root structure and enhancing graft vascularization. Further research is warranted to validate these findings across broader clinical scenarios.

## Introduction

Gingival recession is defined as the apical shift of the gingival margin with respect to the cemento-enamel junction [1]. It is increasingly prevalent with age, particularly in adults over 50 [2]. This condition, linked to significant hard tissue loss, is often associated with periodontal disease, oral hygiene practices, and changes in gingival tissues [3, 4]. While orthodontic treatment can enhance periodontal health, improper use may lead to complications such as tooth mobility, gingival recession, and root resorption. Adult orthodontics now focuses on managing pre-existing recession and at-risk patients. Despite the potential benefits, many patients decline orthodontic interventions due to their duration and high costs. This case report presents an alternative approach to managing gingival recession caused by root protrusion beyond bone boundaries.

## Case presentation

A 28-year-old medically fit female patient presented at the dental care center with severe isolated gingival recession on the lower right central incisor (tooth #41). The patient was a nonsmoker with good oral hygiene and had a history of 5-year orthodontic treatment.

**Figure 1:**
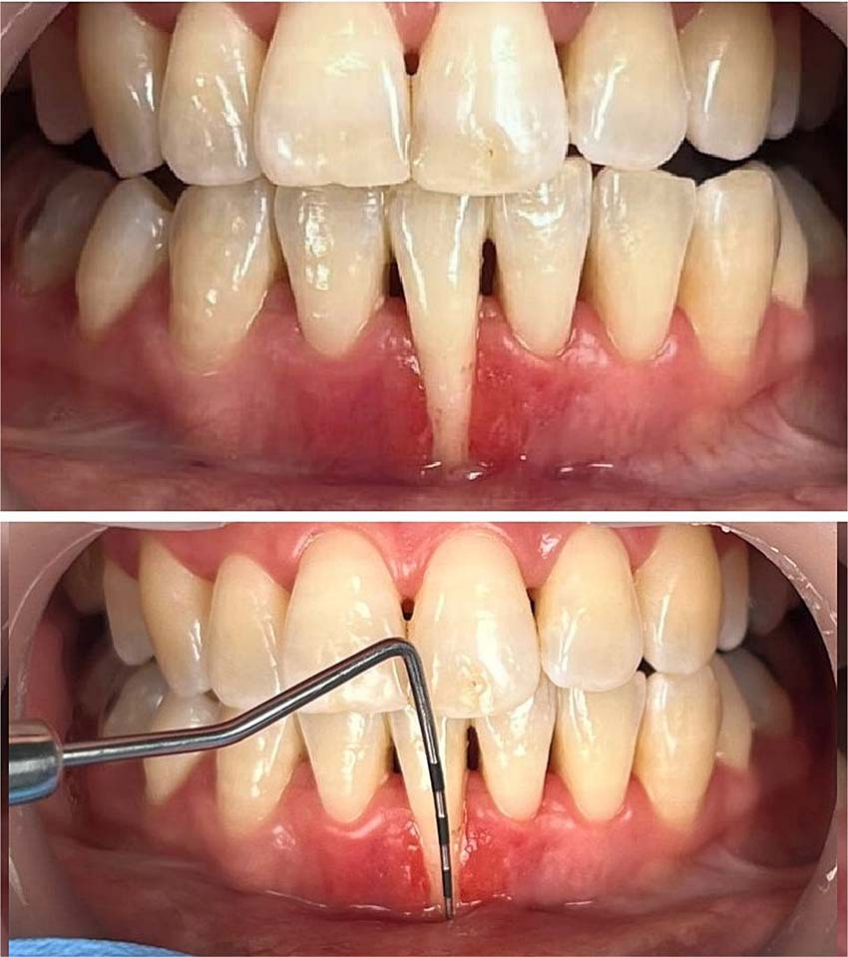
Preoperative clinical status.

The intraoral examination revealed a thin periodontal phenotype with a low full-mouth plaque score. Although there was no excessive tooth mobility, findings included narrow papillae, a shallow vestibule, a probing pocket depth of less than 3 mm, and a recession at the mandibular right central incisor, measuring 8 mm in depth and 4 mm in width. Additionally, no attached or keratinized gingival tissues were identified apical to the defect (Fig. 1).

**Figure 2:**
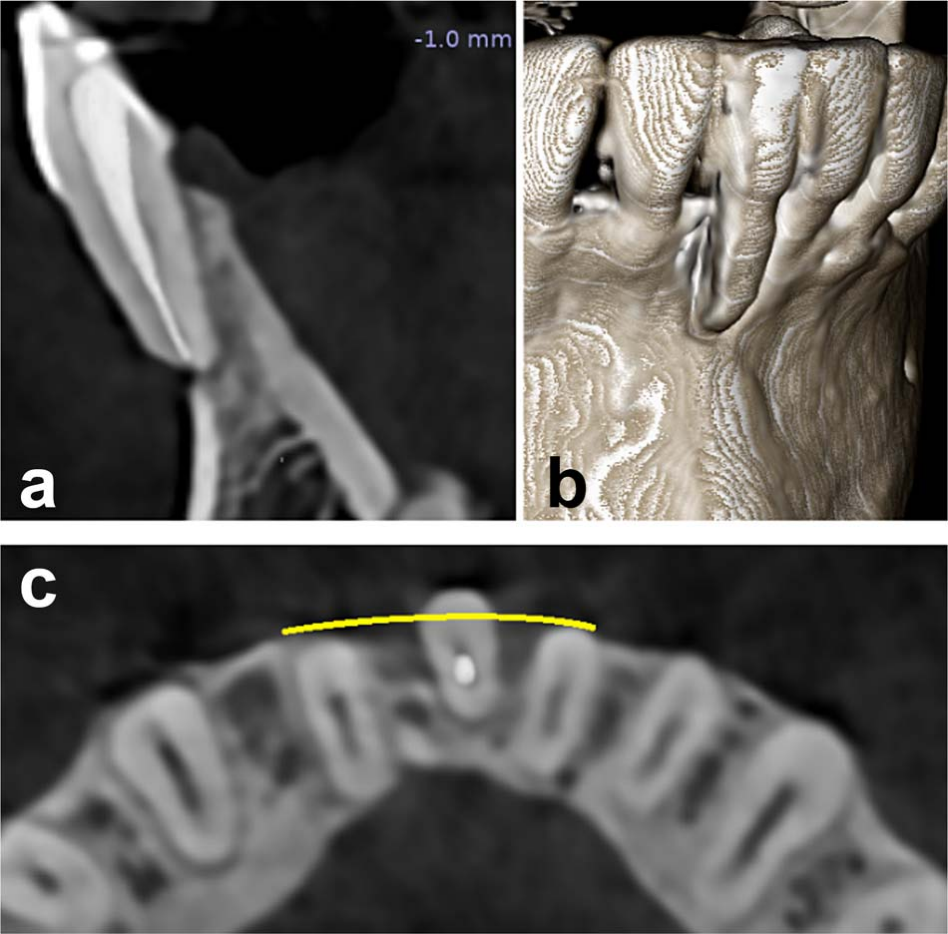
Preoperative radiographical status, (a) sagittal section, (b) 3D construction view, and (c) axial section.

A radiographic examination of the anterior mandibular region revealed no interdental bone loss. However, the affected tooth had previously undergone root canal treatment due to hypersensitivity. CBCT analysis revealed a root protrusion of the affected tooth (Fig. 2a–c).

Based on the clinical and radiological presentation, the gingival recession at the mandibular right central incisor was classified as Miller class III or Cairo Recession Type 2 (RT2) [5, 6]. Following a thorough analysis of the case, it was recommended that the patient undergo orthodontic treatment to reposition the root correctly within the bone. However, the patient categorically refused this plan. Consequently, an alternative approach was considered. It was determined that trimming the protruding part of the root (odontoplasty) without invading the root canal system would be appropriate. Therefore, it was decided to perform an odontoplasty to address the issue and conduct a gingival grafting procedure. This plan was unique in its innovative approach, and the case was closely monitored for 3 months to evaluate the situation.

### Operation

**Figure 3:**
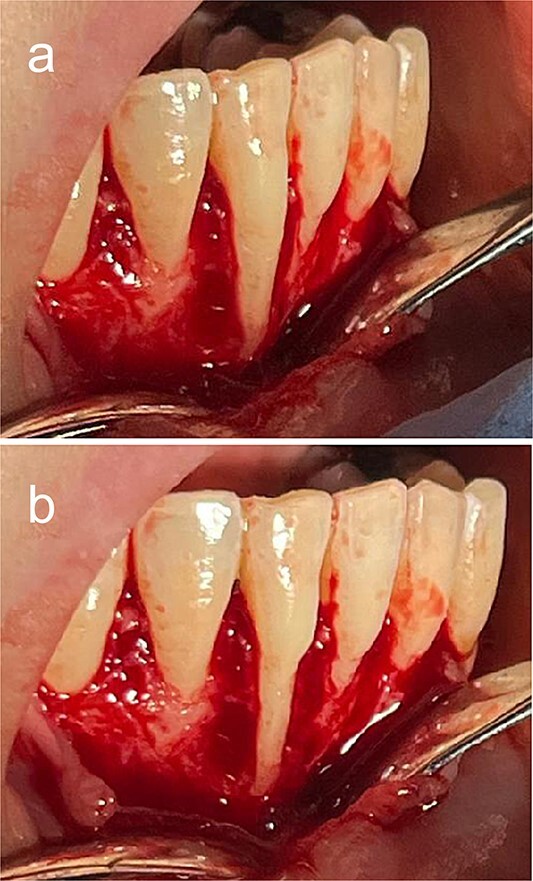
Intraoperative (a) preodontoplasty and (b) postodontoplasty.

A 2% lidocaine with 1:100 000 epinephrine was administered. An intrasulcular incision was made from #43 to #33. A partial-thickness flap was created. The flap was released from any underlying attachment to facilitate flap advancement and closure. The protruding root was trimmed using a large round diamond bur (Fig. 3a and b).

**Figure 4:**
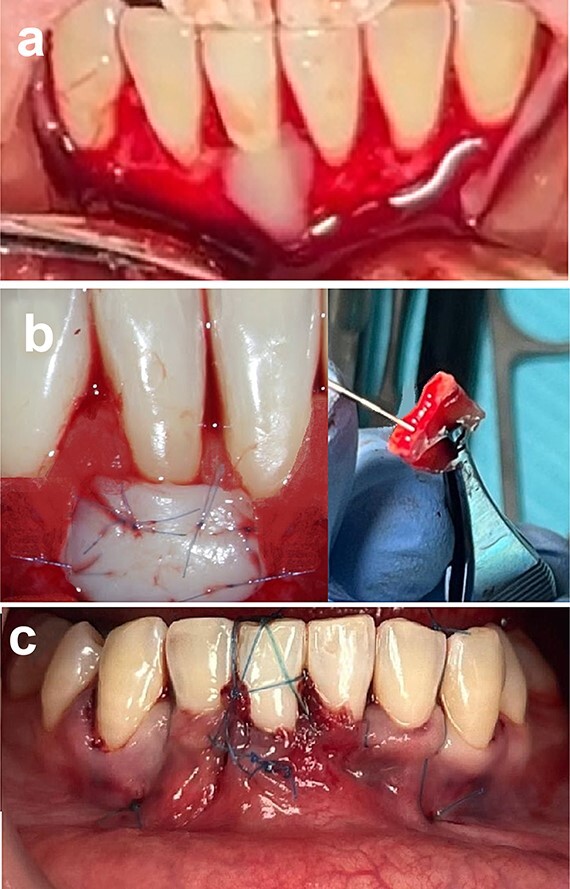
Connective tissue grafting (a) root surface biomodification, (b) connective tissue graft placement, and (c) suturing.

A connective tissue graft (CTG), ~1.5 mm thick, was harvested using a #15C scalpel from the palate. To enhance postoperative comfort and accelerate healing at the palate site, a hemostatic sponge was inserted at the sampling donor site, and the palate was protected with a prefabricated resin plate, worn by the patient for 1 week after the surgery. Following that, the root surface (#41) was biomodified using 24% EDTA gel for 2 minutes. Then, the area was thoroughly rinsed with saline. The CTG was placed and sutured by sling and interrupted sutures using absorbable sutures. Finally, the flap was closed properly and sutured using 4–0 Nylon sutures (Fig. 4a–c).

### Follow-up

**Figure 5:**
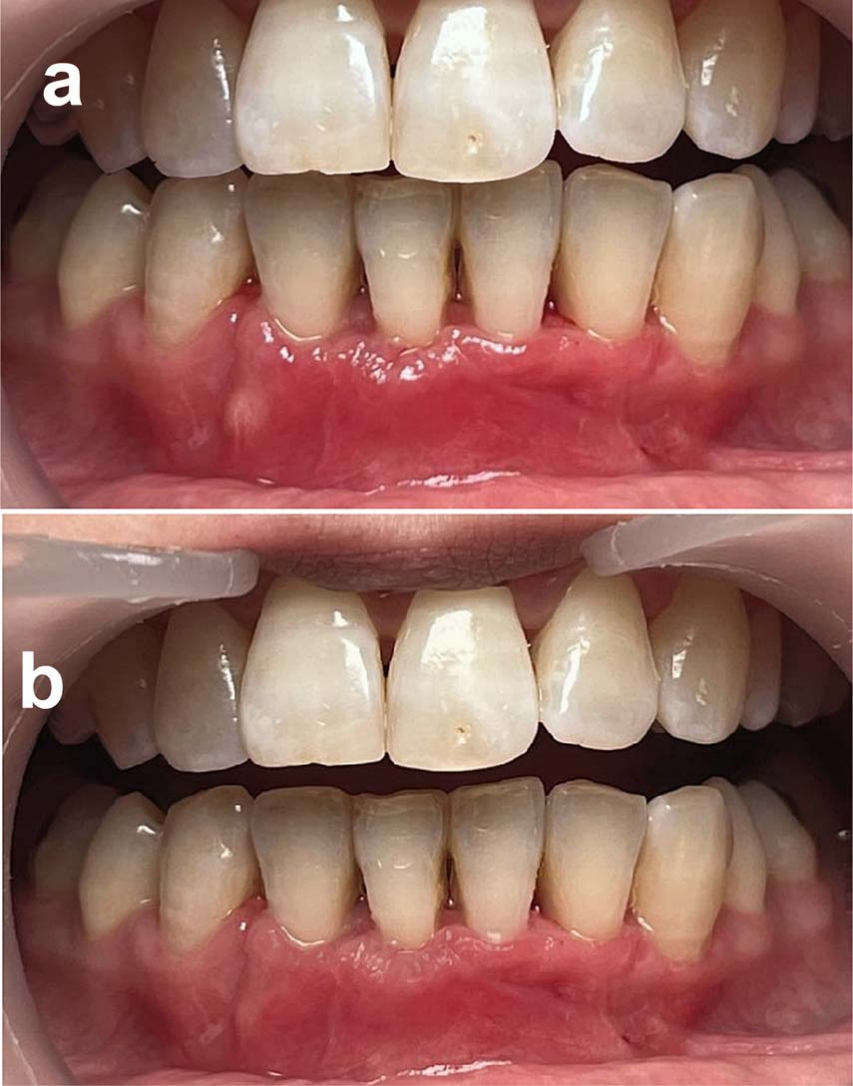
Postoperatively, (a) 2 weeks post-op and (b) 3 months post-op.

A comprehensive follow-up schedule was established, with weekly appointments for the next 3 months. During this period, strict mechanical plaque control measures were implemented using a soft toothbrush. After 3 months, the patient presented with stabilization of clinical results, an increase in the thickness of the keratinized tissue, and complete root coverage at tooth #41, which was achieved. The patient was very satisfied (Fig. 5a and b).

## Discussion

This case highlights a unique approach to managing gingival recession associated with protruded roots beyond the envelope of the alveolar bone. The innovative aspect lies in integrating odontoplasty prior to the soft tissue grafting technique. This sequential strategy represents a novel and effective method for addressing complex cases of gingival recession, providing both functional and esthetic benefits in a simple way [2].

The described technique of removing a portion of root dentine before applying a CTG is not documented in existing literature. Conventional approaches to gingival recession management often focus solely on guided tissue regeneration or gingival grafting techniques [7]. This method emphasizes the importance of odontoplasty as a preparatory step in protruded root cases. A critical consideration in this approach is maintaining the integrity of the root structure by respecting the dentine thickness and avoiding exposure of gutta-percha in endodontically treated teeth. This contrasts with traditional techniques, where minimal alterations to the root surface are typically performed to optimize graft adherence [8, 9].

CTGs for managing gingival recession involve two surgical sites: the primary recipient site at the recession defect and a secondary donor site, typically maxillary palatal tissue. A key characteristic of CTGs is their lack of an independent blood supply, necessitating reliance on the vascular supply from the recipient site for nourishment and survival. For successful integration, it is critical to ensure adequate overlap of the grafted tissue with the surrounding soft tissue at the recipient site, as this facilitates vascularization and stability. Immobilization of the graft at the recipient site is equally essential to prevent displacement and support proper healing [10, 11].

Adequate preparation of the recipient site is paramount to ensure successful graft integration. The prepared area must be large enough to provide sufficient overlap of the donor tissue onto the peripheral recipient bed, typically extending at least 3 mm around the exposed root surface. This overlap ensures an adequate blood supply to the graft, promoting stability and enhancing the likelihood of successful healing [9].

The primary challenge associated with this technique lies in the use of odontoplasty, which requires careful execution to avoid complications. One significant risk is the potential exposure of gutta-percha in endodontically treated teeth, which can compromise the treatment outcome. Additionally, excessive thinning of the root structure during odontoplasty may lead to structural weakening, increasing the likelihood of root fracture under occlusal forces. Therefore, it is essential to consider the overall occlusal load on the recipient site and minimize it as much as possible to ensure long-term stability [12].

This technique is also not suitable for teeth that exhibit pathological mobility. Thinning the dentin in such cases may amplify torque forces on the graft, heightening the risk of graft failure. This limitation underscores the need for further research to better understand the biomechanical implications of this technique and refine patient selection criteria.

## Conclusion

This novel technique, integrating odontoplasty prior to the CTG, has proven effective in managing complex gingival recession cases associated with root protrusion. By maintaining root structure integrity and optimizing graft adherence and vascularization, this method offers significant functional and esthetic benefits. However, further research is needed due to the limited amount of similar cases reported in the literature.

## Data Availability

The data used to support the findings of this study are included within the article.

## References

[1] Cortellini P, Bissada NF. Mucogingival conditions in the natural dentition: narrative review, case definitions, and diagnostic considerations. J Periodontol 2018;89:S204–13. 10.1002/JPER.16-0671. 29926948

[2] Stein JM . Management of gingival recessions: options and prognostic factors. Clin Dent Rev 2019;3:24. 10.1007/s41894-019-0063-7.

[3] Zucchelli G, Mounssif I. Periodontal plastic surgery. Periodontol 2000 2015;68:333–68. 10.1111/prd.12059. 25867992

[4] Imber JC, Kasaj A. Treatment of gingival recession: when and how? Int Dent J 2021;71:178–87. 10.1111/idj.12617. PMC9275303 34024328

[5] Miller PD . Miller classification of marginal tissue recession revisited after 35 years. Compend Contin Educ Dent 2018;39:333–68. 10.1111/prd.12059. 30188152

[6] Fageeh HI, Fageeh HN, Bhati AK, et al. Assessing the reliability of Miller’s classification and Cairo’s classification in classifying gingival recession defects: a comparison study. Medicina (Kaunas) 2024;60. 10.3390/medicina60020205. PMC10890451 38399493

[7] Patel M, Nixon PJ, Chan MFWY. Gingival recession: part 3. Surgical management using free grafts and guided tissue regeneration. Br Dent J 2011;211:353–8. 10.1038/sj.bdj.2011.861. 22015511

[8] Duffy JM, Latimer JM, Fried RM, et al. Minimally invasive coronally advanced flap techniques for correction of gingival recession defects: a review. Compend Contin Educ Dent 2023;44. 10.3390/medicina60020205. 36696277

[9] Indurkar DMS, Sindhuja S, Swathy Krishna J, et al. Gingival recession management using free gingival graft in mandibular anterior region: a case series. Int J Appl Dent Sci 2023;9:07–13. 10.22271/oral.2023.v9.i4a.1841.

[10] Adam K, Staufenbiel I, Geurtsen W, et al. Root coverage using a connective tissue graft with epithelial striation in combination with enamel matrix derivatives - a long-term retrospective clinical interventional study. BMC Oral Health 2019;19. 10.1186/s12903-019-0849-7. PMC6631897 31307447

[11] Lahham C, Ta'a MA. Clinical comparison between different surgical techniques used to manage advanced gingival recession (Miller’s class III & IV). SSRN Electron J 2022;8:e10132. 10.1016/j.heliyon.2022.e10132. PMC9404267 36033300

[12] Rafiuddin S, YG PK, Biswas S, et al. Iatrogenic damage to the periodontium caused by orthodontic treatment procedures: an overview. Dent J 2015;9:228–34. 10.2174/1874210601509010228. PMC4541303 26312093

